# Course of General Fatigue in Patients with Post-COVID-19 Conditions Who Were Prescribed Hochuekkito: A Single-Center Exploratory Pilot Study

**DOI:** 10.3390/jcm14041391

**Published:** 2025-02-19

**Authors:** Kazuki Tokumasu, Nobuyoshi Matsuki, Yuki Otsuka, Yoko Sakamoto, Keigo Ueda, Yui Matsuda, Yasue Sakurada, Hiroyuki Honda, Yasuhiro Nakano, Toru Hasegawa, Ryosuke Takase, Daisuke Omura, Fumio Otsuka

**Affiliations:** 1 Department of General Medicine, Graduate School of Medicine, Dentistry and Pharmaceutical Sciences, Okayama University, Okayama 700-8558, Japanfumiotsu@md.okayama-u.ac.jp (F.O.); 2 Department of Epidemiology, Medical School, Okayama University, Okayama 700-8558, Japan; 3 Center for Innovative Clinical Medicine, Okayama University Hospital, Okayama 700-8558, Japan; 4 Clinical & Educational Center for Kampo Medicine, Okayama University Hospital, Okayama 700-8558, Japan

**Keywords:** fatigue assessment scale (FAS), general fatigue, hochuekkito, kampo medicine, long COVID, post-COVID-19 condition

## Abstract

**Background**: After the start of the COVID-19 pandemic, general fatigue in patients with long COVID and post-COVID-19 conditions (PCC) became a medical issue. Although there is a lack of evidence-based treatments, Kampo medicine (traditional Japanese medicine) has gained attention in Japan. At an outpatient clinic in Japan specializing in long COVID, 24% of all prescriptions were Kampo medicines, and 72% of Kampo medicine prescriptions were hochuekkito. However, there has been no prospective, quantitative study on the course of fatigue in patients with long COVID and PCC who were prescribed hochuekkito. The aim of this study was to clarify the course of fatigue in those patients. **Methods**: This study included patients aged 18 years or older with general fatigue who visited the long COVID specialized outpatient clinic at Okayama University Hospital and consented to participate after being prescribed hochuekkito. We reviewed the backgrounds of the patients, and we evaluated the patients’ fatigue assessment scale in person or online. **Results**: Twenty patients were enrolled in this study from September to December in 2023. The average age of the patients was 42.9 years (SD: 15.8 years) and 12 patients (60%) were female. After hochuekkito administration, the fatigue assessment scale score decreased from 35.9 (SD: 5.9) at the initial visit to 31.2 (SD: 9.4) after 8 weeks, indicating a trend for improvement in fatigue (difference: 4.7; 95% CI: 0.5–8.9). **Conclusions**: A trend for improvement in fatigue was observed in patients with long COVID and PCC who were prescribed hochuekkito, indicating a potential benefit of hochuekkito for general fatigue in such patients. General fatigue in patients with long COVID or PCC can be classified as post-infectious fatigue syndrome and is considered a condition of qi deficiency in Kampo medicine, for which hochuekkito is appropriately indicated.

## 1. Introduction

After detection of the first cases in December 2019, COVID-19 caused a global epidemic with more than 776 million infections and more than 7 million deaths [1]. In May 2023, the World Health Organization issued a statement that the COVID-19 emergency for global health had ended and that control of persistent infections was important [2,3]. Management of the sequelae of COVID-19 is also an important component of healthcare since many cases of long COVID and post-COVID-19 conditions (PCC) have been reported [4,5]. A systematic review of the prevalence of long COVID showed that the prevalence of persistent symptoms after COVID-19 illness varied from 0% to 93%, with a pooled estimate of 42.1%. More than 50 symptoms in patients with long COVID and PCC has been reported, and general fatigue was shown to be the most common symptom with incidences ranging from 58% to 64% [5,6,7]. A major concern is that some patients have symptoms that persist for more than two years [8]. General fatigue has been reported to be the most frequent symptom in patients with long COVID and PCC worldwide and in Japan [9,10]. General fatigue is an important symptom due to its potential to persist for more than six months and its adverse effects on daily life [11,12].

A previous study in patients with long COVID and PCC who had general fatigue showed that the most frequently prescribed Kampo medicine was hochuekkito [13]. Hochuekkito was shown to be effective for improving the quality of life in elderly patients in a double-blind, randomized control trial [14] and it was shown to be effective for improving general fatigue in patients receiving chemotherapy for cancer [15,16]. It has also been reported that Kampo medicine including hochuekkito might be effective for treating long COVID symptoms, especially general fatigue [17]. In a study conducted in Korea, Bojungikki-tang, which has the same ingredients as those in Japanese hochuekkito, was administered to 15 patients with long COVID. After 12 weeks of treatment with the herbal medicine, fatigue symptoms were significantly improved in 80% of the patients [18].

However, the efficacy of hochuekkito for improving general fatigue in patients with long COVID and PCC remains unknown. We therefore conducted a single-arm exploratory pilot study with the aim of confirming improvements in fatigue symptoms in patients with long COVID and PCC who received hochuekkito. This study serves as a preliminary step toward the design of placebo-controlled randomized trials.

## 2. Methods

### 2.1. Study Design and Setting

This study was designed as a single-center exploratory pilot study conducted at a COVID-19 aftercare clinic. The aim of this single-arm pilot study was to confirm observable improvements in fatigue symptoms following administration of the Kampo medicine hochuekkito in patients with long COVID and post-COVID conditions (PCC) as a preliminary step toward evaluating the potential effects of hochuekkito.

On 15 February 2021, a COVID-19 aftercare clinic (CAC) was established within the Department of General Medicine at Okayama University Hospital, a tertiary care facility with 865 beds located in western Japan. The CAC was established to evaluate and manage patients experiencing long COVID and PCC. The clinic accepts patients who have had sequelae for at least one month (four weeks) following the onset of COVID-19 as well as patients referred to it from other medical facilities.

### 2.2. Patients’ Inclusion and Definitions of Long COVID and PCC

This study included patients aged 18 years or older who had long COVID or PCC with general fatigue who visited the CAC at Okayama University Hospital during the period from September to December in 2023. We selected patients who fulfilled the inclusion criteria and did not fulfill the exclusion criteria (Table 1) and who consented to participate in this study.

PCC is an umbrella term used by the US Centers for Disease Control and Prevention (CDC) that encompasses a range of signs, symptoms, and conditions that are present for at least 4 weeks after SARS-CoV-2 infection [20]. Long COVID is defined as an infection-associated chronic condition that occurs after SARS-CoV-2 infection and with symptoms that are present for at least 3 months as a continuous, relapsing and remitting, or progressive disease state that affects one or more organ systems [21].

The sample size for this study was set at 20 cases. This study was designed as an exploratory single-arm pilot study. Therefore, the sample size was not determined using hypothesis testing based on prior studies. Instead, the sample size was set according to the number of feasible cases. As a reference, in a practical clinical setting, assuming a mean difference in the fatigue assessment scale (FAS) scores with a threshold mean of 1.0, an expected mean of 3.5, and a standard deviation of 4.0, the required sample size would be 18 cases, with a one-sided significance level of 0.05 and a power of 0.8. While this calculation was not used for determining the sample size, it was considered as a reference point during the study design.

**Figure 1:**
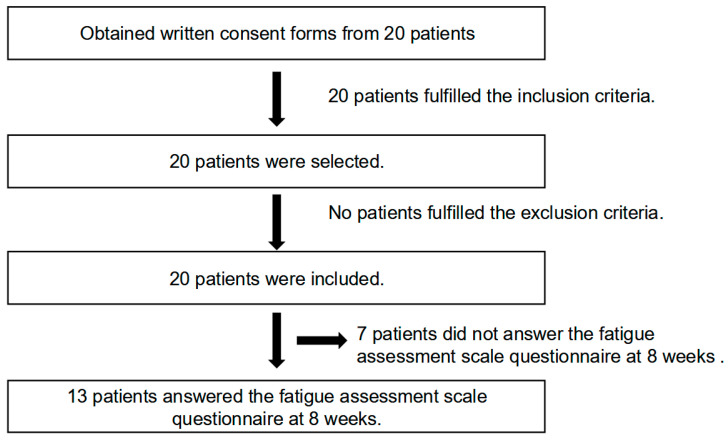
Schematic diagram detailing participant selection based on the inclusion and exclusion criteria. We included 20 patients initially and received data for 13 patients who answered the fatigue assessment scale questionnaire at 8 weeks which were then used for analysis.

A schematic diagram detailing participant selection based on the inclusion and exclusion criteria is shown in Figure 1.

### 2.3. Intervention

Patients were administered hochuekkito, a Kampo medicine, for 8 weeks. Hochuekkito has 10 herbal ingredients, including Astragalus Root and Atractylodes Rhizome which have been reported to have anti-fatigue and immunomodulatory properties. The prescribed dosage was 7.5 g/day, divided into two or three doses. Hochuekkito was supplied by two manufacturers, JUNKOU (Osaka, Japan) and TSUMURA (Tokyo, Japan), based on the availability of formulations at the clinic. This treatment was part of routine clinical care for patients with long COVID and was conducted as a prospective observational study.

### 2.4. Data Collection

Clinical information including information on age, gender, severity of the acute phase of COVID-19 [22], duration from the onset of COVID-19 to the initial visit to the CAC, history of COVID-19 vaccination, and clinical symptoms of long COVID was obtained from medical records.

### 2.5. Dose of Extract of Hochuekkito

Hochuekkito was prescribed when physicians determined it to be clinically necessary on the basis of results from clinical examinations. Kampo medicine (including prescription of hochuekkito) is covered by Japanese health insurance. The two products, JUNKOU Hochuekkito and TSUMURA Hochuekkito, are widely available in Japan.

(1) JUNKOU Hochuekkito [23]

The daily dose of this product, 7.50 g, contains 4.90 g of the dried extract (Hochuekkito extract) from the following mixed crude drugs.
JP Ginseng4.00 gJP Jujube2.00 gJP Atractylodes Rhizome4.00 gJP Bupleurum Root2.00 gJP Astragalus Root4.00 gJP Glycyrrhiza1.50 gJP Japanese Angelica Root3.00 gJP Ginger0.50 gJP Citrus Unshiu Peel2.00 gJP Cimicifuga Rhizome1.00 g(JP: Japanese pharmacopeia)

Inactive ingredients include corn starch and lactose hydrate.

(2) TSUMURA Hochuekkito [24]

7.5 g of TSUMURA Hochuekkito extract granules (hereafter TJ-41) contains 5.0 g of a dried extract of the following mixed crude drugs.
JP Astragalus Root4.0 gJP Atractylodes Lancea Rhizome4.0 gJP Ginseng4.0 gJP Japanese Angelica Root3.0 gJP Bupleurum Root2.0 gJP Jujube2.0 gJP Citrus Unshiu Peel2.0 gJP Glycyrrhiza1.5 gJP Cimicifuga Rhizome1.0 g(JP: Japanese pharmacopeia)

Inactive ingredients include JP magnesium stearate and JP lactose hydrate.

### 2.6. Instruments and Outcomes

We used the Japanese version of the fatigue assessment scale (FAS) [25] for assessment of outcomes. The original version of the FAS was developed for assessment of fatigue in the general population [26]. The 10-item self-report questionnaire has been validated for sarcoidosis, a disease manifesting as fatigue [27,28]. In the questionnaire, one out of five answer categories, from never to always, is chosen (1: never, 2: sometimes (about monthly or less), 3: regularly (a few times a month), 4: often (about weekly), and 5: always (about every day)). The total FAS score is calculated by summing the scores for all questions (questions 4 and 10 having reverse scores). The total score ranges from 10 to 50, with a higher score indicating worsening fatigue. A four-point change in the FAS score is regarded as clinically significant, representing the minimal clinically important difference. [29]. A PDF and digital version of the FAS in each language can be found on the website of the ild care foundation [30]. The reliability and validity of the Japanese version of the FAS have been demonstrated [25].

We evaluated the primary endpoint using the Japanese version of the FAS at 8 weeks after starting the administration of hochuekkito. The period of 8 weeks was based on our clinical experience indicating that it takes 8 weeks for general fatigue to improve. A previous study also showed that the median time to recovery from fatigue was 63 days (95% CI: 47–181 days) according to Kaplan–Meier curves of the symptoms [31], and we therefore considered the period of 8 weeks to be a reasonable observation period. We also evaluated the FAS score at 1 week, 2 weeks, and 4 weeks after the start of intervention as secondary endpoints. These evaluations were conducted using paper or electronic versions of the FAS, with participants responding directly when they visited the clinic for the baseline and at 8 weeks and by an online system via email for weeks 1, 2, and 4.

### 2.7. Statistical Analyses

First, we examined descriptive statistics for characteristics of the subjects. Second, we calculated the point estimate and 95% confidence interval for the difference between the mean of the FAS at baseline and the mean of the FAS at the primary endpoint (8 weeks). Subjects with missing values at the baseline and at week 8 were excluded from the analysis, even if data were available for other weeks. Subjects with baseline and week 8 data were excluded only for the week with missing values. All statistical analyses were performed using Stata/SE 18.0 (StataCorp, College Station, TX, USA).

### 2.8. Ethical Approval

For ethical considerations, informed consent from all patients was obtained. This study was approved by the Ethics Committee of Okayama University Hospital (No. 2305-015, approval date: 28 April 2023), and the study adhered to the Declaration of Helsinki.

## 3. Results

Twenty patients with long COVID or PCC were included in this study. The average age of the patients was 42.9 years (standard deviation (SD): 15.8 years) and 12 patients (60%) were female (Table 2). All of the participants were prescribed JUNKO Hochuekkito or TSUMURA Hochuekkito (contents shown in Section 2.4.).

**Figure 2:**
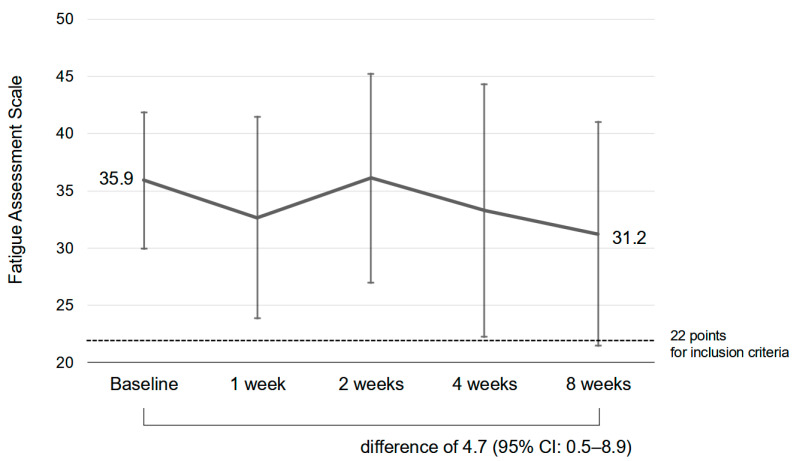
Mean and standard deviation scores of the Japanese version of the fatigue assessment scale at 1, 2, 4, and 8 weeks from baseline. Higher scores indicate greater fatigue. The score for the Japanese version of the FAS decreased from 35.9 (SD: 5.9) at the initial visit to 31.2 (SD: 9.4) after 8 weeks, showing a trend for improvement in fatigue (difference of 4.7: 95% CI: 0.5–8.9).

The scores for the Japanese version of the FAS decreased from 35.9 (SD: 5.9) at the initial visit to 31.2 (SD: 9.4) after 8 weeks (difference of 4.7: 95% CI: 0.5–8.9). Figure 2 shows the mean and standard deviation scores of the FAS at 1, 2, 4, and 8 weeks from the baseline. Mean (SD) scores were 32.7 (8.8) at 1 week, 36.1 (9.1) at 2 weeks, and 33.3 (11.0) at 4 weeks. Seven subjects with missing baseline or week 8 data were excluded. Of the thirteen eligible subjects, seven with missing data at 1 week, four with missing data at 2 weeks, and three with missing data at 4 weeks were excluded only for the week in which they were missing data (validated numbers of questionnaires: *n* = thirteen (baseline), *n* = six (1 week), *n* = nine (2 weeks), *n* = ten (4 weeks), and *n* = thirteen (8 weeks)). All patients were treated at an outpatient setting and no patients required hospitalization.

## 4. Discussion

General fatigue in patients with long COVID or PCC who were prescribed hochuekkito was evaluated by using scores for a fatigue scale (the Japanese version of the FAS). The scores decreased, indicating improvement in general fatigue. According to the minimal clinical improvement of fatigue, a decrease in the FAS score of 4 is required [29]. At 8 weeks after prescription of hochuekkito, the FAS score had decreased by 4.7 (95% CI: 0.5–8.9). This indicated improvement of general fatigue in the clinical setting.

Hochuekkito, used in this study, is one of the Kampo medicines that are administered for treatment of various symptoms, including general fatigue [32]. In contrast to modern medicine based on pathophysiology and treatment, Kampo medicine focuses on the concept that diseases arise from the collapse of balances and harmony between organ systems and the external environment [32]. Additionally, the absence or deficiency of the body’s vital energy, known as qi, may lead to the manifestation of illness [33]. General fatigue after acute infection is often identified to be the status of “qi deficiency” [34,35], and hochuekkito is acknowledged as a prominent formula in Kampo medicine for compensating ‘qi’. Qi is the universal energy that exists in the world. In Kampo medicine, it is considered that all living organisms are condensed forms of qi. Deficiency of qi represents decline of vital activity. In that stage, the patient has listlessness, fatigue, and loss of energy and appetite [36]. ICD-11 defined a qi deficiency pattern characterized by decreased vitality, fatigue, weakness, appetite loss, shortness of breath, no desire to speak, spontaneous sweating, and feeble pulse. That pattern may be explained by a decreased or insufficient quantity of qi [37].

Pharmacological studies have shown that hochuekkito has immune stimulatory effects against infections and cancer, immunomodulatory effects against allergies and certain inflammatory diseases, and ameliorative effects against exhaustion and frailty. Hochuekkito has been shown to enhance locomotor activity in normal mice and mice with chronic fatigue syndrome induced by injection of the Brucella abortus antigen [38,39]. Hochuekkito also showed efficacy in animal models of depression [40] and epilepsy [41]. The effects of hochuekkito may be due to its influence on serotonin levels and the nervous system [35]. Among the ingredients in hochuekkito, Ginsenoside, one of the main constituents of Ginseng, has been reported to have anti-fatigue, anti-stress, and tonic effects and it is conventionally used to improve qi deficiency [42]. Astragalus Root was also reported to improve fatigue and malaise [42].

## 5. Limitations

This study has several limitations. Firstly, the subjects of this study were patients from a single clinic specializing in long COVID or PCC that only accepts referrals from other clinics. Consequently, more severe cases may have been included, potentially introducing a selection bias and leading to an underestimation of the observed improvements in fatigue. Furthermore, the small sample size may have further restricted the generalizability of the findings to the target population. Secondly, there may be confounding factors, such as gender and the time from infection to the initial clinic visit that could have influenced the results. Although gender has been suggested to be a potential confounder in similar studies on fatigue [43,44], the small sample size and unbalanced gender distribution (nine males and four females) in this study made statistical adjustment or stratified analysis impractical. Similarly, the duration between infection and the initial visit was not adjusted for due to the limited sample size. These factors may have introduced confounding effects that cannot be entirely ruled out. Further study with a comparison of two groups (hochuekkito group and control group) is required to examine the effectiveness of hochuekkito. There is a plan to conduct a randomized controlled trial for these two groups [45]. Thirdly, seven patients had missing FAS data at the 8-week mark, which was the main outcome. One patient reported improvement in fatigue and could return to work, but the patient’s FAS data could not be obtained (No. 2). Missing data for a patient who dropped out due to improvement in fatigue might lead to an underestimation of the main results. Fourthly, general fatigue symptoms associated with long COVID and PCC are known to improve over time [10,31]. Since this study was a single-group observational study, some patients may have experienced a natural improvement in fatigue, rather than improvement solely due to the medication. Fifthly, the FAS data for the three patients (No. 3, 6, and 16) may not be purely the course of taking hochuekkito. This is because the Kampo medicine may have been changed to another Kampo medicine prescription or another Kampo medicine prescription may have been added for these patients during the 8 weeks (observational period of this study). Finally, the study included cases for which more than one year had passed since the onset of symptoms (No. 3, 5, 11, 13, 15), suggesting that symptoms might have become chronic, similar to the symptoms of myalgic encephalomyelitis/chronic fatigue syndrome.

## 6. Conclusions

A trend towards improvement in fatigue was observed in patients with long COVID or PCC who were prescribed hochuekkito, indicating a potential benefit of hochuekkito for general fatigue in such patients. General fatigue in patients with long COVID or PCC can be classified as a post-infectious fatigue syndrome and is considered a condition of qi deficiency in Kampo medicine, for which hochuekkito is appropriately indicated.

## Figures and Tables

**Table 1:** Inclusion and exclusion criteria.

	Inclusion Criteria (Patients Fulfilling the Following Conditions Were Included).
1	Patients with COVID-19 confirmed by PCR or other tests and at least one week after onset of illness.
2	Patients with a score for the Japanese version of the fatigue assessment scale (FAS) of 22 or more at the time of the visit.
3	Patients who are able to answer the questionnaire online via smartphone, computer, etc., and who are able to answer the questionnaire in person.
4	Patients aged 18 years or older at the time of obtaining the consent form.
5	Patients prescribed Hochuekkito as a therapeutic agent.
	Exclusion criteria
1	Patients with any of the following diseases or laboratory abnormalities found in examinations at the time of the first visit (the following diseases or laboratory abnormalities possibly indicating general fatigue): Active infectious diseases (active pulmonary tuberculosis, active infectious diseases such as hepatitis B and C viruses, etc.) Endocrine and metabolic disorders such as adrenal insufficiency, thyroid dysfunction, and diabetes mellitus. Hypokalemia, hypo/hypernatremia, and hypercalcaemia.
2	Patients who were taking Kampo medicines other than Hochuekkito at the first visit.
3	Women who are pregnant or have the possibility of pregnancy.
4	Patients who are currently attending a psychiatry department and are being treated for disorders of ‘ICD-10 (International Classification of Diseases) Chapter 5: Mental and Behavioural Disorders’ [19].

PCR: polymerase chain reaction, ICD: International Classification of Diseases.

**Table 2:** Participants’ Characteristics.

	Number (%) *n* = 20
**Gender**	
Female	12 (60.0)
Male	8 (40.0)
**Age** (mean)	42.9 (25–57)
18–30 years	6 (30.0)
31–50 years	8 (40.0)
>51 years	6 (30.0)
**Vaccination**	
0–1 dose	5 (25.0)
More than two doses	15 (75.0)
**Duration after the onset of COVID-19 to the first visit**	
Total duration (months)	5.3 (1.5–13.5)
Less than one month	1
1–2 months	6
2–3 months	2
More than 3 months	9

## Data Availability

The datasets used and/or analyzed in this study are included in the Supplementary Table S1. Further inquiries can be directed to the corresponding author upon reasonable request.

## Supplementary Materials

The following supporting information can be downloaded at: https://www.mdpi.com/article/10.3390/jcm14041391/s1, Table S1: Total data for the Japanese version of FAS.

## Abbreviations

coronavirus disease 2019 (COVID-19); COVID-19 aftercare clinic (CAC); the US Centers for Disease Control and Prevention (CDC); 95% confidence interval (95% CI); International Classification of Diseases (ICD); myalgic encephalomyelitis/chronic fatigue syndrome (ME/CFS); post-COVID-19 condition (PCC); polymerase chain reaction (PCR); standard deviation (SD)
