# Supplementary material for: Course of General Fatigue in Patients with Post-COVID-19 Conditions Who Were Prescribed Hochuekkito: A Single-Center Exploratory Pilot Study
Source: J Clin Med. 2025 Feb 19;14(4):1391. doi: 10.3390/jcm14041391 (PMC11857072; doi:10.3390/jcm14041391)
Supplement: Supplementary file 1 [file jcm-14-01391-s001.zip › jcm-3424766-supplementary.pdf]

Supplementary Materials: Table S1; Total data for the Japanese version of FAS

Number of participants	Baseline	1 week	2 weeks	4 weeks	8 weeks
1	24	18	18	15	13
2	41	37	45	39	
3	35		38	37	32
4		25			
5	31	32			32
6	39	45			41
7	35	32	38		44
8	38	38			
9	37		38	37	
10	36	32	32	32	33
11	35	40	40	43	
12	40	37			
13	41	46	43	46	
14	46		39	39	37
15	41		44	44	38
16	40			43	30
17	42		50	46	36
18	32			19	14

19	37	37	37	36	36
20	29		29	22	20

The blanks indicate missing values.
